# Diagnostic Accuracy of Intraoperative Frozen Section Examination of Pelvic Lymph Nodes in Cervical Carcinoma: A Cross-Sectional Study in Eastern India

**DOI:** 10.7759/cureus.111520

**Published:** 2026-06-25

**Authors:** Shaily Srivastava, Sunil Kumar Mahto, Manoj K Paswan, Anshu Jamaiyar

**Affiliations:** 1 Pathology, Rajendra Institute of Medical Sciences, Ranchi, IND

**Keywords:** cervical cancer, diagnostic accuracy, frozen section, histopathology, lymph node metastasis, pelvic lymph nodes

## Abstract

Background

Cervical cancer continues to be a major cause of cancer-related incidence and mortality in women, especially within low- and middle-income countries where available screening tests and preventive healthcare services are suboptimal. Intraoperative frozen section examination (IFSE) enables real-time evaluation of suspicious pelvic lymph nodes and helps guide surgical management. The present study was undertaken to evaluate the diagnostic accuracy and reliability of IFSE of pelvic lymph nodes in patients with cervical carcinoma using final histopathology as the reference standard.

Methods

Our hospital-based cross-sectional study was conducted in the Department of Pathology in collaboration with the Department of Obstetrics and Gynecology at Rajendra Institute of Medical Sciences, Ranchi, India, over a period of 18 months. A total of 80 patients with histopathologically confirmed cervical carcinoma undergoing pelvic lymph node dissection were included. Suspicious lymph nodes were submitted for frozen section examination and compared with final paraffin-embedded histopathology. Diagnostic indices, including sensitivity, specificity, positive predictive value, negative predictive value, and diagnostic accuracy, were calculated.

Results

The mean age was 47.89 ± 13.95 years, with the highest proportion of patients in the 51-60-year age group. Frozen section examination demonstrated metastatic positivity in 37 (46.3%) patients, whereas final histopathological examination identified nodal metastasis in 60 (75%) cases. IFSE demonstrated sensitivity of 60%, specificity of 95%, positive predictive value of 97.3%, negative predictive value of 44.2%, and overall diagnostic accuracy of 68.8%. Frozen section positivity showed a significant association with human papillomavirus (HPV) infection, smoking history, parity, and advancing age.

Conclusion

Frozen section analysis was found to have high specificity and positive predictive value, thus being highly reliable for the diagnosis of nodal metastasis during surgery and for intraoperative decision-making. Frozen section analysis of pelvic lymph nodes can be considered as a useful adjunctive tool in the surgical management of cervical carcinoma, especially in low-resource settings.

## Introduction

Cervical cancer continues to be a major cause of cancer-related incidence and mortality in women, especially within low- and middle-income countries where available screening tests and preventive healthcare services are suboptimal [1,2]. Cervix uteri cancer is one of the most important malignancies affecting women worldwide, and developing countries account for a disproportionate share of the disease burden and associated deaths [1,2]. Persistent infection with high-risk human papillomavirus (HPV), particularly HPV-16 and HPV-18, is a well-established primary cause of cervical carcinogenesis [3,4]. Other risk factors, including multiparity, smoking, immunosuppression, early sexual debut, and co-infection with other sexually transmitted infections, also contribute to the malignant progression of the disease [5,6]. Lymphatic spread is considered one of the most important prognostic determinants in carcinoma cervix [7,8]. In patients undergoing radical hysterectomy with pelvic lymphadenectomy, accurate intraoperative identification of lymph node involvement is essential for therapeutic decision-making and surgical planning, as nodal metastasis is associated with increased recurrence rates, poor overall survival, and the need for adjuvant chemoradiation therapy [9].

Histopathological examination of paraffin-embedded tissue sections remains the gold standard for diagnosing nodal metastasis; however, definitive reports are usually unavailable during surgery. Intraoperative frozen section examination (IFSE) has therefore emerged as a rapid diagnostic adjunct that enables real-time evaluation of suspicious pelvic lymph nodes and helps guide surgical management, particularly in avoiding unnecessary radical procedures in patients with metastatic nodal disease [10]. Several studies have demonstrated high specificity of frozen section analysis for detecting nodal metastases in cervical cancer. However, variability persists regarding sensitivity, negative predictive value, and overall diagnostic concordance with final histopathology, especially in resource-limited tertiary care centers where technical expertise and infrastructure may differ [11-13]. In addition, limited Indian data are available evaluating the clinicopathological correlates of frozen section positivity and its usefulness in real-time intraoperative decision-making.

Considering the prognostic significance of pelvic lymph node metastasis and the potential role of IFSE in guiding surgical management, the present study was undertaken to evaluate the diagnostic accuracy and reliability of IFSE of pelvic lymph nodes in patients with cervical carcinoma using final histopathology as the reference standard. The study also aimed to assess the association between frozen section findings and relevant clinicopathological parameters and to determine its usefulness as a real-time diagnostic tool in a tertiary care center.

## Materials and methods

Our hospital-based cross-sectional study was conducted in the Department of Pathology in collaboration with the Department of Obstetrics and Gynecology at Rajendra Institute of Medical Sciences (RIMS), Ranchi, India, over a period of 18 months from July 2024 to January 2026. Ethical clearance was obtained from the Institutional Ethics Committee of RIMS (IEC No. 90, dated February 01, 2025), and written informed consent was obtained from all participants before enrollment. Female patients with histopathologically confirmed cervical carcinoma undergoing surgical management with pelvic lymph node dissection were included in our study. Patients with inflammatory cervical lesions, low-grade squamous intraepithelial lesions (LSIL), high-grade squamous intraepithelial lesions (HSIL), carcinoma in situ, and those unwilling to participate were excluded.

Sample size was calculated using the formula:

$N = \frac{4PQ}{d^{2}}$,

where P represented expected sensitivity, Q = 100 − P, and d represented allowable error.

Based on the pooled sensitivity reported in a recent systematic review evaluating frozen section examination in cervical cancer, the minimum calculated sample size was 80 patients [14]. Consecutive eligible patients fulfilling the inclusion criteria were enrolled during our study period.

Pelvic lymph node dissection, including obturator, internal iliac, and external iliac lymph nodes, was performed intraoperatively under general anesthesia. Suspicious lymph nodes identified on visualization and palpation were submitted fresh for frozen section examination and transported immediately to the pathology laboratory without fixative. Representative tissue sections were processed using a cryostat maintained between −18°C and −24°C. Tissue was embedded in Leica tissue-freezing medium, sectioned at approximately 3 µm thickness, stained with rapid hematoxylin and eosin stain, and examined microscopically for metastatic deposits.

The rest of the tissue was then routinely processed for paraffin-embedded histopathology, considered the gold standard. All clinical and demographic data, such as age, parity, HPV infection status, smoking history, other sexually transmitted infections, frozen section finding, and final histopathological diagnosis, were documented on a structured proforma. The statistical analysis was done using Statistical Package for the Social Sciences (SPSS) software. All continuous variables were presented as mean ± SD, and all categorical variables were presented as frequencies and percentages. Diagnostic indices, including sensitivity, specificity, positive predictive value, negative predictive value, and diagnostic accuracy, were calculated. Chi-square test, independent t-test, and Cohen’s kappa statistics were used for analysis, and a p-value < 0.05 was considered statistically significant.

## Results

A total of 80 patients with histopathologically confirmed cervical carcinoma were included in our study. The mean age was 47.89 ± 13.95 years, with the highest proportion of patients in the 51-60-year age group (21; 26.3%). HPV infection was present in 37 (46.3%) patients, while multiparity was observed in 52 (65%) patients. Smoking history and other sexually transmitted infections were documented in 26 (32.5%) and 56 (70%) patients, respectively (Table 1).

**Table 1:** Baseline demographic and clinicopathological characteristics of the study population (n = 80) HPV: Human papillomavirus.

Variable	Category	N	Percentage
Age group (years)	11–20	1	1.3%
	21–30	10	12.5%
	31–40	14	17.5%
	41–50	19	23.8%
	51–60	21	26.3%
	61–70	11	13.8%
	71–80	4	5.0%
HPV infection	Present	37	46.3%
	Absent	43	53.7%
Parity	Nulliparous	10	12.5%
	Primiparous	18	22.5%
	Multiparous	52	65.0%
Smoking history	Present	26	32.5%
	Absent	54	67.5%
Other sexually transmitted infections	Present	56	70.0%
	Absent	24	30.0%

Frozen section examination demonstrated metastatic positivity in 37 (46.3%) patients, whereas final histopathological examination identified nodal metastasis in 60 (75%) patients (Table 2).

**Table 2:** Distribution of intraoperative frozen section and final histopathological findings

Diagnostic modality	Positive, n (%)	Negative, n (%)
Frozen section examination	37 (46.3%)	43 (53.7%)
Final histopathology	60 (75.0%)	20 (25.0%)

Comparison between frozen section examination and final histopathology revealed 36 true-positive, 19 true-negative, 1 false-positive, and 24 false-negative cases. A statistically significant association was observed between frozen section findings and final histopathology (χ² = 18.253, p < 0.001) (Table 3).

**Table 3:** Concordance between frozen section examination and final histopathology Chi-square value = 18.253; p-value = <0.001.

Frozen section result	Histopathology positive	Histopathology negative	Total
Positive	36 (45.0%)	1 (1.3%)	37 (46.3%)
Negative	24 (30.0%)	19 (23.7%)	43 (53.7%)
Total	60 (75.0%)	20 (25.0%)	80 (100%)

IFSE demonstrated a sensitivity of 60%, specificity of 95%, positive predictive value of 97.3%, negative predictive value of 44.2%, and overall diagnostic accuracy of 68.8%. Agreement analysis showed fair concordance between frozen section examination and final histopathology (κ = 0.398, p < 0.001) (Table 4).

**Table 4:** Diagnostic performance of intraoperative frozen section examination Cohen’s kappa (κ) = 0.398; p-value = <0.001.

Diagnostic parameters	Value (%)
Sensitivity	60.0%
Specificity	95.0%
Positive predictive value (PPV)	97.3%
Negative predictive value (NPV)	44.2%
Diagnostic accuracy	68.8%

Frozen section positivity showed a significant association with HPV infection (χ² = 7.011, p = 0.008), smoking history (χ² = 14.578, p < 0.001), parity (χ² = 22.800, p < 0.001), and advancing age (p < 0.001). Positive frozen section findings were more common among multiparous women (34; 65.4%) and smokers (20; 76.9%). No significant association was observed between other sexually transmitted infections and frozen section positivity (p = 0.304) (Table 5).

**Table 5:** Association between frozen section positivity and clinicopathological variables *Chi-square test; **Unpaired t-test. HPV: Human papillomavirus; STI: Sexually transmitted infection.

Variable	Frozen section positive, n (%)	Frozen section negative, n (%)	Statistical test (df)	p-value
HPV infection	23 (62.2%)	14 (37.8%)	7.011 (1)*	0.008
Smoking history	20 (76.9%)	6 (23.1%)	14.578 (1)*	<0.001
Other STI	28 (50.0%)	28 (50.0%)	1.056 (1)*	0.304
Parity				
Multiparous	34 (65.4%)	18 (34.6%)	22.800 (2)*	<0.001
Primiparous	3 (16.7%)	15 (83.3%)		
Nulliparous	0 (0.0%)	10 (100%)		
Age (Mean ± SD)	55.86 ± 11.54	41.02 ± 12.16	5.574**	<0.001

## Discussion

The mean age at diagnosis was 47.89 ± 13.95 years, with the 51-60-year age group showing the highest frequency of 21 (26.3%) patients. Both Sung et al. [1] and Arbyn et al. [2] reported similar age distributions. Multiparity was seen in 52 (65%) patients, which agrees with the results obtained by Muñoz et al. [5], and a smoking history was noted in 26 (32.5%) patients, as reported by Plummer et al. [6] in relation to the effect of tobacco exposure in the development of cervical cancer. Final histopathology showed pelvic lymph node metastasis in 60 (75%) cases, and IFSE showed positivity in 37 (46.3%) cases. This discrepancy underscores the fact that frozen section analysis is an inadequate means of detecting micrometastases and low-volume metastatic deposits. This was also found by Bats et al. [15] and Balaya et al. [16], who showed that the sensitivity of the frozen section examination was decreased by occult metastatic disease only seen on permanent paraffin sections and ultrastaging.

Our study demonstrated high specificity, similar to that found in previous studies assessing IFSE in cervical cancer. Scholz et al. [11] reported that it had excellent specificity and was useful for intraoperative detection of macrometastatic nodal disease. The frozen section diagnosis proved to be of great value to the surgeons for making decisions regarding cervical cancer surgery, as shown by Ferrandina et al. [12] and Fanfani et al. [13]. IFSE had high specificity (95.2%) in the current study but low sensitivity and negative predictive value (44.2%). In a systematic review and meta-analysis of the utility of frozen section analysis in cervical cancer, Agustí et al. [14] found that sensitivity ranged from 40% to 80%, whereas specificity was consistently high.

The relatively low sensitivity (60%) and negative predictive value (44.2%) observed in our study deserve particular consideration. These findings indicate that a substantial proportion of metastatic lymph nodes may remain undetected during intraoperative assessment, resulting in false-negative diagnoses. Possible explanations include sampling error, tissue-freezing artifacts, limited tissue evaluation during rapid intraoperative processing, and the inability of routine frozen section examination to reliably detect micrometastases or isolated tumor cells. Consequently, although a positive frozen section result is highly reliable, a negative result should be interpreted with caution and should not exclude the possibility of metastatic disease. These findings reinforce the importance of permanent paraffin-embedded histopathological examination as the definitive diagnostic standard for nodal assessment in cervical carcinoma.

HPV infection, smoking, parity, and increasing age were significantly associated with lymph node metastatic involvement as detected by frozen section examination. The mean age of patients with positive frozen section was significantly higher than that of patients with negative frozen section (55.86 ± 11.54 vs. 41.02 ± 12.16 years; p < 0.001). HPV infection and smoking were also factors independently associated with the presence of nodes in the positivity, which lends support to the pathogenetic mechanisms proposed by Bosch et al. [3] and Doorbar et al. [17]. Our study underscores the potential utility of IFSE as a live diagnostic tool during surgery for cervical carcinoma. Positive frozen section diagnosis may contribute to the early planning of adjuvant therapy for the patient and intraoperative adjustment of the surgical therapy. Nevertheless, the diagnosis of negative frozen section should be taken with a grain of salt, as metastatic disease can still be identified on permanent histopathology.

The strengths of our study are that histopathology was used as the gold standard, and clinicopathological correlation was studied in a real-life tertiary care setting. The limitations of our study include its single-center design, relatively small sample size, and the absence of ultrastaging or immunohistochemical examination for the detection of micrometastases. In addition, multivariable analysis was not performed; therefore, potential confounding among clinicopathological variables could not be accounted for, and the reported associations should be interpreted as unadjusted findings from our study. Because frozen section examination was primarily performed on clinically suspicious lymph nodes, the prevalence of nodal metastasis observed in this study may be higher than that seen in unselected cervical cancer populations, potentially limiting the generalizability. Furthermore​​​​​​, the cross-sectional design and the absence of multivariable regression analysis precluded adjustment for potential confounding among age, HPV infection, smoking status, and parity.

## Conclusions

The frozen section analysis was found to have high specificity and positive predictive value, thus being highly reliable for the diagnosis of nodal metastasis during surgery and intraoperative decision-making. The moderate sensitivity and low negative predictive value indicate that metastatic deposits, particularly low-volume disease and micrometastases, may be missed during intraoperative assessment. Therefore, negative frozen section findings should be interpreted cautiously and confirmed by permanent histopathological examination, which remains the gold standard for nodal evaluation. In conclusion, frozen section analysis of pelvic lymph nodes can be considered as a useful adjunctive tool in the surgical management of cervical carcinoma.
